# Geographical Clustering and Environmental Determinants of Schistosomiasis from 2007 to 2012 in Jianghan Plain, China

**DOI:** 10.3390/ijerph15071481

**Published:** 2018-07-13

**Authors:** Yingnan Niu, Rendong Li, Juan Qiu, Xingjian Xu, Duan Huang, Yubing Qu

**Affiliations:** 1 Key Laboratory of Monitoring and Estimate for Environment and Disaster of Hubei Province, Institute of Geodesy and Geophysics, Chinese Academy of Sciences, Wuhan 430077, China; niuyingnan16@mails.ucas.ac.cn (Y.N.); qiujuan@asch.whigg.ac.cn (J.Q.); huangduan@asch.whigg.ac.cn (D.H.); quyubing15@mails.ucas.ac.cn (Y.Q.); 2 College of Earth and Planetary Sciences, University of Chinese Academy of Sciences, Beijing 100049, China; 3 Hubei Provincial Center for Disease Control and Prevention, Wuhan 430079, China; xuxj8412@foxmail.com

**Keywords:** schistosomiasis, clustering, environment determinants, Jianghan Plain, China

## Abstract

This study compared changes in the spatial clustering of schistosomiasis in Jianghan Plain, China by applying Kulldorff’s spatial scan statistic. The Geodetector software was employed to detect the environmental determinants of schistosomiasis annually from 2007 to 2012. The most likely spatial cluster in 2007 covered the north-central part of Jianghan Plain, whereas those observed from 2008 to 2012 were toward the south, with extended coverage in generally the same areas across various periods, and some variation nevertheless in precise locations. Furthermore, the 2007 period was more likely to be clustered than any other period. We found that temperature, land use, and soil type were the most critical factors associated with infection rates in humans. In addition, land use and soil type had the greatest impact on the prevalence of schistosomiasis in 2009, whereas this effect was minimal in 2007. The effect of temperature on schistosomiasis prevalence reached its maximum in 2010, whereas in 2008, this effect was minimal. Differences observed in the effects of those two factors on the spatial distribution of human schistosomiasis were inconsistent, showing statistical significance in some years and a lack thereof in others. Moreover, when two factors operated simultaneously, a trend of enhanced interaction was consistently observed. High-risk areas with strong interactions of affected factors should be targeted for disease control interventions.

## 1. Introduction

Schistosomiasis, an acute and chronic disease caused by parasitic flatworms referred to as schistosomes, is endemic in about 75 developing countries. It mainly affects people who live in rural agricultural and suburban areas, with significant economic and public health consequences [1,2]. The number of people suffering from schistosomiasis worldwide is approximately 252 million, and it is estimated that 4400–200,000 people die from the disease each year [3]. Within recent years, schistosomiasis has been successfully controlled in many countries. Since China’s national government has actively promoted the elimination of schistosomiasis, control efforts have made considerable progress in recent decades, followed by a considerable reduction in the infection rates. Surveillance and analysis data of schistosomiasis in China for 2015 showed that the total infection rate of the resident population maintained a steady decline. However, owing to the complex objective factors of prevalence and transmission as well as the existence of risk factors associated with repeated and rising epidemics, it is necessary to reinforce the importance of prevention and monitoring strategies regarding schistosomiasis [4,5,6].

The survival, reproduction, and spread of schistosomes within a region are closely related to the conditions that facilitate the survival and reproduction of schistosomiasis pathogens and parasitic hosts [7]. First, the activities of miracidia and cercariae in the life history of schistosomes are directly related to water temperature, wind speed, and sediment content. Second, the growth and reproduction of schistosomes have an important relationship with the intermediate host snails. The spatial distribution of *Oncomelania* snails is related to various environmental factors, such as surface vegetation, beach groundwater level, beach soil moisture content, soil moisture content, surface relief, climatic conditions, altitude elevation, microenvironmental physicochemical factors, hydraulic factors like water flow speed (which will block floating debris to which snails attach and effectively prevent snails from spreading), physical and chemical conditions of soil, and several other environmental factors [8,9,10,11,12].

The epidemiology of schistosomiasis infection in China shows unique spatial characteristics. Based on the geographical patterns of endemic areas and the complex ecological features of the sole intermediate host snails of schistosomes, the epidemic areas of schistosomiasis in China can be divided into three types: lake and marshland regions, plain regions with networks, and hilly and mountainous regions [13,14]. The lakes and marshes of Hubei Province, a predominantly epidemic region, comprise the main area of interest in the middle and lower reaches of the Yangtze River in southern China. Previous studies have described a relatively severe epidemic in Jianghan Plain, located in the central and southern parts of Hubei Province [4].

Few studies have evaluated changes in the spatial distribution of human schistosomiasis and the impact of and variations in factors that promote the transmission of schistosomiasis in Jianghan Plain. In the present study, we employed spatial and space–time scan statistics to analyze the clusters and number of people infected with schistosomiasis. Furthermore, the Geodetector software was used to assess the environmental risks to human schistosomiasis rates in Jianghan Plain from 2007 to 2012.

## 2. Materials and Methods

### 2.1. Study Area

Jianghan Plain, which is located at latitude 29°26′–31°10′ N, longitude 111°45′–114°16′ E, spans roughly 30,000 km^2^ in the middle and southern parts of Hubei Province. It forms the main part of the middle and lower reaches of the Yangtze River, covering eight counties and municipalities, including Jingzhou District, Shashi District, Jiangling County, Gong’an County, Jianli County, Shishou City, Honghu City, Songzi City, and three provincial directly managed cities: Xiantao City, Qianjiang City, and Tianmen City. Jianghan Plain is situated in the middle subtropical region of China, where the climate is warm and humid. The rain and heat occur during the same period, and a dense river network is present, comprising numerous lakes and vast areas of water. Thus, this area satisfies the breeding and propagation conditions of the unique intermediate snail hosts of schistosomes. Based on the aforementioned factors, the epidemic of schistosomiasis is rather critical within the study area.

### 2.2. Parasitological Data

**Figure 1:**
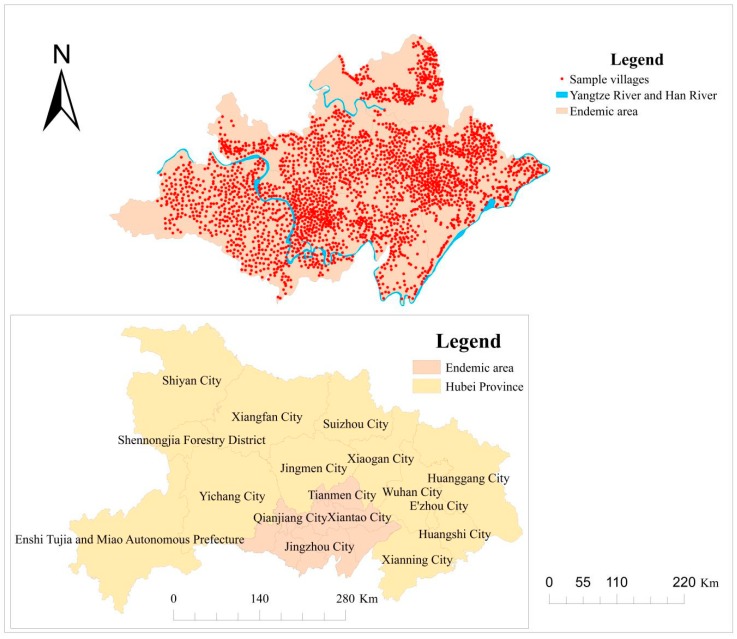
Locations of study area and sample villages.

Data on schistosomiasis prevalence, including the names, number of infected people, population of endemic villages, and human infection rates from 2007 to 2012, were obtained from the Institute of Schistosomiasis Control, Hubei Provincial Center for Disease Control and Prevention. We collected village-level vector geographic data and completed 10 vector maps for each county (city) by registering, splicing, and encoding each village. The “Join” function in the ArcGIS software was then used to correlate village-level human disease data with the village-level vector geographic map [15,16]. In addition, the central coordinates of each administrative village were calculated to generate the points of the schistosomiasis epidemic villages in Jianghan Plain (Figure 1).

### 2.3. Environmental Data

Environmental data regarding land use, soil type, soil texture (sand percentage, silt percentage, and clay percentage), and water data in Jianghan Plain was provided by the Data Center for Resources and Environmental Sciences, Chinese Academy of Sciences (RESDC) (http://www.resdc.cn). It should be noted that since the RESDC does not have year-by-year land use data, the land use data of 2010 was used instead. Although it is considered somewhat rough, it can still explain some associated challenges.

In addition, all 8-day global 1-km products for land surface temperature at daytime (LSTD) and at night (LSTN), as well as the monthly global products for the normalized difference vegetation index (NDVI) covering Jianghan Plain for each year, were downloaded from the Level-1 and Atmosphere Archive and Distribution System (https://ladsweb.modaps.eosdis.nasa.gov/sear). We used the natural break method to divide numerical data, such as temperature, NDVI, and soil texture, into five, five, and four categories, respectively. Each category was then encoded for convenience. The MODIS Reprojection Tool (MRT, https://lpdaac.usgs.gov/tools/modis_reprojection_tool) was then used to project, mosaic, and specify a geographic subset. Land use, soil type, soil texture, average LSTD, average LSTN, average TDN (average day and night temperatures), average NDVI, and DER (distances from the endemic villages to the river) of each endemic village were extracted using the ArcGIS software.

### 2.4. Statistical Analysis

#### 2.4.1. Cluster Analysis

We adopted spatial and space–time scan statistics of the number of infected individuals in each village from 2007 to 2012 to analyze the temporal and spatial clustering of schistosomiasis.

Spatial cluster analysis: Annual spatial cluster analysis applying the discrete Poisson model was performed with the Martin Software for spatial, temporal, and space–time scan statistics (SaTScan software, version v9.6, https://www.satscan.org/). The maximum spatial cluster size was set to 50% of the population at risk, and a circular window shape was used to detect clusters. The minimum number of cases in clusters with high rates was limited to at least 10. In addition, the replication number was set at 999 (default = 999).

Space–time cluster analysis: Space–time analysis was applied to scan for clusters with high risk using the Space–Time Permutation model and the SaTScan software. The maximum spatial cluster size was set to 50% of the population at risk and the window shape was circular. The minimum and maximum temporal cluster sizes were set to 1 year and 50% of the study period, respectively. The minimum number of cases in clusters with high rates was set to 2. In addition, the number of replications was defined as 999.

#### 2.4.2. Detection of Geographical Environment Factors on Schistosomiasis

Geodetector software (http://www.geodetector.org/), developed by Dr. Xu and Prof. Wang, was used to analyze the environmental factors of schistosomiasis. This method is based on spatial variation analysis of the geographical strata of variables used to assess the environmental risks to human health. It consists of four parts: the risk detector indicates where the risk areas are located; the factor detector identifies which factors are responsible for the risk; the ecological detector reveals the relative importance of the factors; and the interaction detector reveals whether the risk factors interact with each other or independently lead to disease [17,18]. Wang and Hu [17] offer a detailed explanation of the principle behind the geographical detector. We used the following three detectors to reveal the characteristics and influencing mechanisms of schistosomiasis in Jianghan Plain from 2007 to 2012.

Factor detection: This detector was used to determine whether a geographical factor stratum was responsible for an observed spatial pattern of schistosomiasis. The main idea was to compare the accumulated dispersion variance of each subregion with the dispersion variance of the entire study region. The smaller the ratio, the stronger the disease contribution of the stratum, which can be measured, as follows [19]:

(1) $$q=1-\frac{N_{D1}Var_{D1}+N_{D2}Var_{D2}+N_{D3}Var_{D3}}{N\times Var_{D}}$$

where *N* and *N_Di_* denote the number of units in schistosomiasis layer G and strata *D_i_* in the factor layer *D*, respectively; *Var_Di_* denotes the variance in infection rates of human schistosomiasis in each strata; *Var_D_* denotes the variance in infection rates of human schistosomiasis over the entire study area A.

If factor D completely controls schistosomiasis, it would be equal to 1; however, if it is completely unrelated to schistosomiasis, it would be equal to 0. Therefore, values would lie within the range [0, 1]. The larger the value, the greater the impact of factor D on schistosomiasis prevalence. This value, therefore, can be used to quantify the association between schistosomiasis prevalence and the risk factors studied.

Interaction detection: Interaction detection was developed to identify whether two health determinants, when considered together, weaken or enhance each other or are independent in the development of the disease. The main idea of this process is to compare the sum of the disease contributions of two individual attributes vs. the contribution of the two attributes when considered together.

Ecological detection: Comparison of the variance calculated from divisions of each subregion based on a particular determinant, with that based on another determinant, was the general idea of this detector. Based on this theory, we compared whether one risk factor is more significant than another in controlling the spatial patterns of schistosomiasis.

## 3. Results

### 3.1. Spatial Cluster Analysis

**Figure 2:**
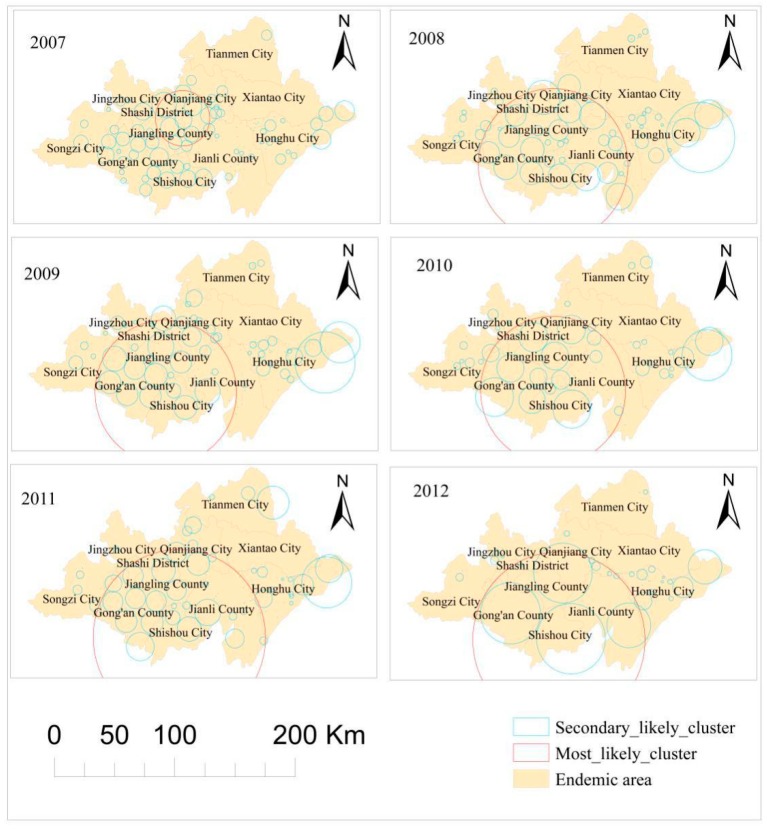
Spatial clusters detected by utilizing the discrete Poisson model from 2007 to 2012 in Jianghan Plain.

The results of spatial cluster analysis are presented in Figure 2. Table 1 indicates several essential spatial characteristics of the number of people affected by schistosomiasis. First, the most likely spatial cluster in 2007 covered the southwestern part of Qianjiang City, the southeastern area of Shashi District, and most areas of Jiangling County. Moreover, spatial coverage of the most likely clusters in 2008, 2009, 2010, 2011, and 2012 were identical and extended to the southwestern region of Qianjiang City and the vast majority of areas of Shashi District, Jiangling County, Jianli County, Shashi District, and Gong’an County. In addition to these most likely clusters, several secondary likely clusters were also identified. Furthermore, as shown in Table 1, the number of clusters declined from 109 in 2007 to 42 in 2012, with fluctuations along the way. In addition, changes observed in the number of endemic villages in the most likely clusters could have been divided into four phases as increasing from 2007 to 2008 and 2009 to 2011, decreasing from 2008 to 2009, and remaining unchanged from 2011 to 2012.

**Figure 3:**
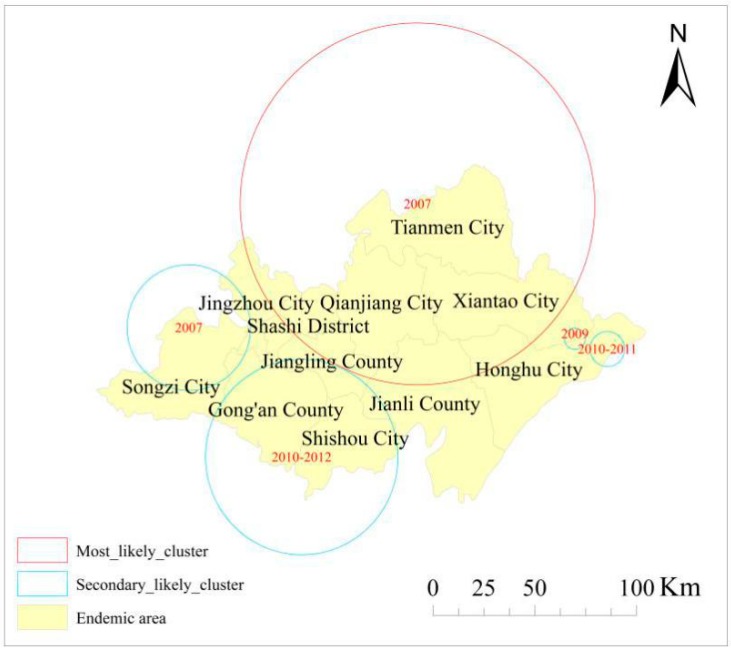
Space–time clusters detected using the Space–Time Permutation model.

Space–time cluster analysis of human schistosomiasis cases: Retrospective space–time analysis of clusters is presented in Figure 3. Table 2 indicates that five clusters were detected in Jianghan Plain from 2007 to 2012. The log likelihood ratio (LLR) ranged from a maximum of 1404.203 to a minimum 82.678, with a *p*-value less than 0.001. This suggests the significance of both space and time in these areas. The most likely cluster occurred in 2007 and consisted of 1014 endemic villages distributed among most of the northern Jianghan Plain, covering Tianmen City, Xiantao City, Qianjiang City, and Shashi District, as well as partial areas of Honghu City, Jianli County, Jiangling County, and Jingzhou City (LLR = 1404.203, *p* < 0.001). In addition, four secondary space–time clustered areas were detected: one in 2007, covering 116 villages in most of the northern parts of Songzi City and a small part of western Jingzhou District and Gong’an County; the second space–time cluster was mainly distributed in a small area in northeast Jiangling County and most parts of Gong’an County, Shishou City, and Jiangling County, covering 500 endemic villages from 2010 to 2012; the third cluster covered 23 endemic villages in the period from 2010 to 2011, covering a small area in northeast Honghu City; and the fourth cluster was located in a tiny area of four villages along the border between Honghu City and Xiantao City in 2009.

### 3.2. Environmental Factor Analysis

#### 3.2.1. Factor Detector

The results of factor detector analysis (Table 3) revealed that the environmental factors associated with the infection rates of human schistosomiasis from 2007 to 2012 were ranked by their contribution to schistosomiasis. In 2007 and 2012, the most influencing factors were average TDN, average LSTN, and land use. In 2008 and 2009, the top three factors included land use, soil type, and average LSTN. In 2010, the average LSTN, average LSTD, and land use were the essential factors in controlling the infection rates of human schistosomiasis. In 2011, the important factors included the average LSTN, land use, and average TDN.

Based on these analyses from 2007 to 2012, it is evident that among the environmental factors selected in this study, temperature, land use type, and soil type had the greatest impact on schistosomiasis in Jianghan Plain. The other factors under consideration had a relatively weak impact on the prevalence of the disease. The effects of land use and soil types on schistosomiasis increased from 2007 to 2009, decreased from 2009 to 2012, and reached its maximum in 2009. Furthermore, the effect of temperature on schistosomiasis was reduced from 2007 to 2008, increased from 2008 to 2010, decreased from 2010 to 2012, and reached its maximum in 2010.

#### 3.2.2. Ecological Detector

**Figure 4:**
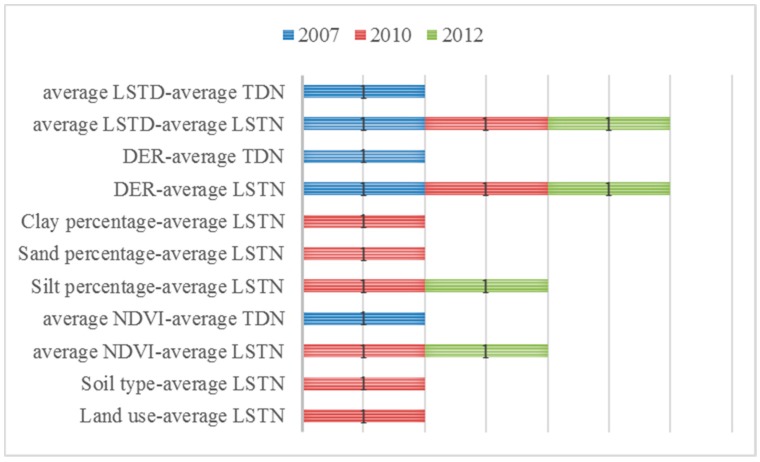
Statistical significance of q-value between different risk factors. Average NDVI, average normalized different vegetation index; DER, distance from the endemic villages to the river; average LSTD, average land surface temperature at daytime; average LSTN, average land surface temperature at night; average TDN, average day and night temperatures. 1 indicates significant differences between the two risk factors, with 95% confidence.

We determined whether the effects of the two factors on the spatial distribution of infection rates of human schistosomiasis were significantly different and conducted tests using the ecological detector. The results are presented in Figure 4, where years not shown indicate that the differences between any two factors were not statistically significant. First, in 2007, the differences between average NDVI and average TDN, DER and average TDN, average LSTD and average TDN, DER and average LSTN, and average LSTD and average LSTN were all significant. Second, in 2008, 2009, and 2011, the differences between any two factors were not statistically significant. Third, in 2010, the differences between average LSTN and any other factors excepting for average TDN showed statistical significance. Apart from those differences, no other significant differences were noted in 2010. In 2012, the differences between the average NDVI and average LSTN, silt percentage and average LSTN, DER and average LSTN, and average LSTD and average LSTN were all significant.

The differences observed in the effects of two factors on the spatial distribution of human schistosomiasis were inconsistent, showing significance in some years and a lack thereof in others.

#### 3.2.3. Interaction Detector

**Figure 5:**
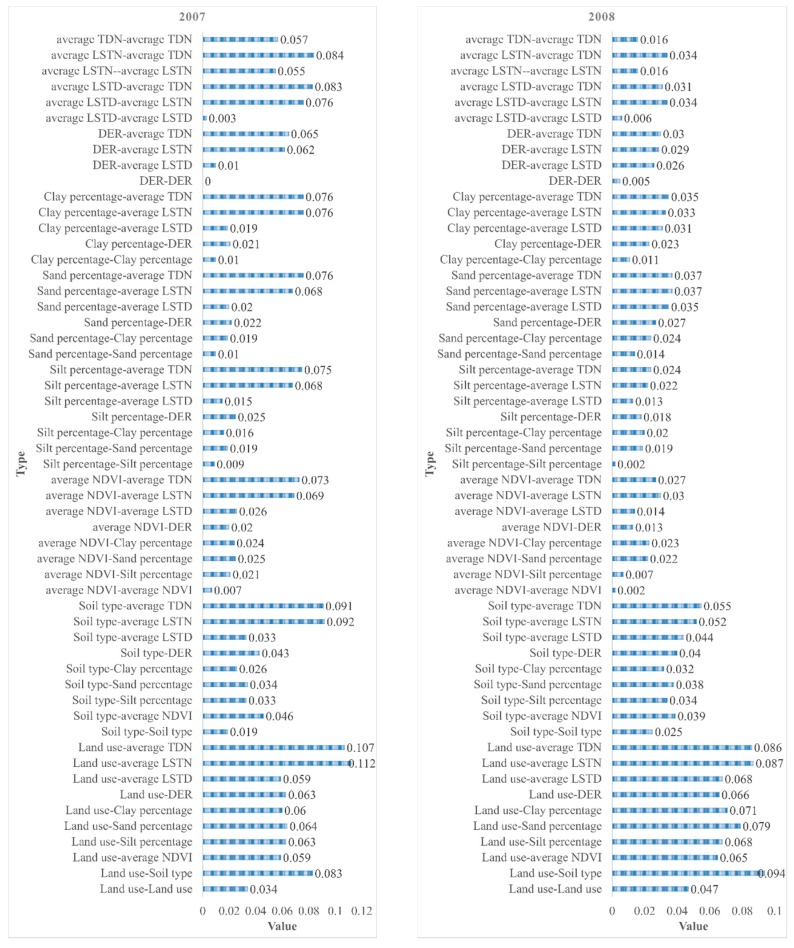

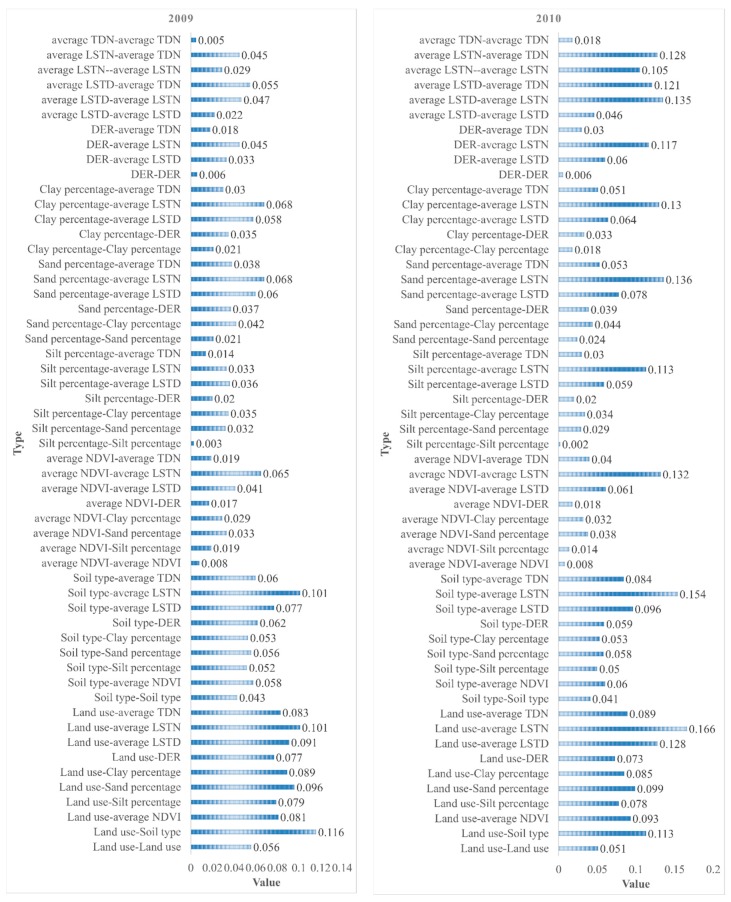

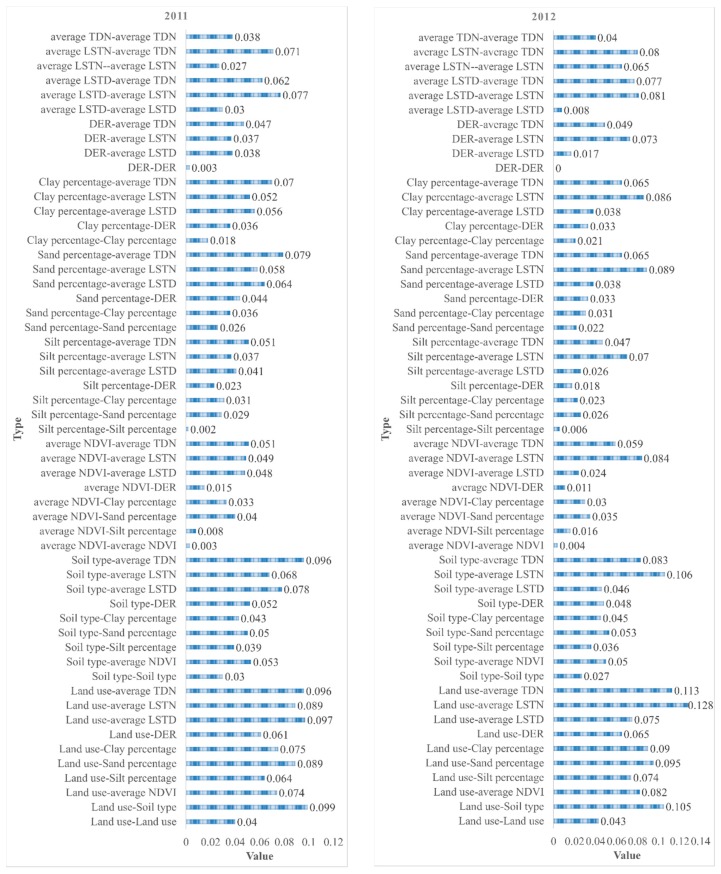
Results of analysis of the interaction detectors. Average NDVI, average normalized different vegetation index; DER, distance from the endemic villages to the river; average LSTD, average land surface temperature at daytime; average LSTN, average land surface temperature at night; average TDN, average day and night temperatures.

Interaction detection was applied to check whether two schistosomiasis determinants work independently or not. The outcomes are presented in Figure 5. We considered land use and soil type in 2007 as an example to interpret the results. These two determinants accounted for 3.4% and 1.9% of schistosomiasis rates, respectively. However, the joint effect of the two factors was 8.3%. Thus, land use and soil type operating together enhance the effects of each other in the control of schistosomiasis. Based on the analysis of Figure 5, we found that whenever any two factors operated together, a trend of enhanced interaction was observed.

## 4. Discussion

Understanding the spatial distribution of schistosomiasis is of great importance in the control of schistosomiasis [20]. Jianghan Plain is widely known as a susceptible area of schistosomiasis in China, owing to its climate and geographical characteristics [4,7,21,22]. This study presents the application of spatial, temporal, and space–time scan statistics as well as Geodetector analysis of the changing spatiotemporal patterns and environmental factors of schistosomiasis in Jianghan Plain in the middle and southern parts of Hubei Province, China during the period 2007–2012.

Spatial, temporal, and space–time scan statistics (SaTScan statistics) perform well when used for the geographical surveillance of disease to detect spatial or space–time disease clusters and determine statistical significance [23]. These statistics have also been applied in numerous previous studies [15,24,25,26]. Several conclusions can be drawn using this method. First, the purely spatial cluster analysis of large populations of schistosomiasis infections, using the Kulldorff’s spatial scan statistic, showed that the number of clusters and relative risk declined but fluctuated. However, coverage of the most likely clustered areas showed a dynamic and increasing trend. These findings are mainly due to a lower number of infected people within recent years than in the past. In addition, the mobility of the population has shown a significant increase. Furthermore, the prevalence of schistosomiasis was relatively low and scattered, resulting in a wide range of clusters.

Since 2004, schistosomiasis prevention and control in China has included the implementation of a comprehensive strategy based on controlling the source of infection. This has promoted a trend of recovery from the disease and diminished the epidemic of schistosomiasis to a relatively low level [20]. To promote schistosomiasis prevention efforts for the province even further, active exploration of the optimal prevention and control strategies have been outlined to achieve the second phase of the “planning” targets for the control of transmission. The Hubei Provincial People’s Government and the former Ministry of Health and Ministry of Agriculture reached a consensus at the end of 2008 to support schistosomiasis prevention efforts in Hubei Province and agreed to launch a joint schistosomiasis prevention campaign from 2009 to 2013 [27].

Furthermore, space–time cluster analysis indicated the presence of five clusters: two in 2007, two during the periods 2010–2012 and 2010–2011, and one in 2009, respectively. It is worth noting that no cluster was observed for 2008. Since 2006, Hubei Province has implemented prevention and control strategies based on the control of infectious sources and establishment of new rural construction projects in severely endemic areas. The strategies include combining resources from multiple sectors of the society, concentrating on governance of severely affected villages, replacing cattle with machinery, prohibiting beach grazing, water and toilet reform, human and livestock inspection and treatment, examination of snails, and health education, among other measures that have achieved favorable effects [28].

However, certain challenges associated with implementation of the measures to “eliminate farming cattle and replace cattle with machinery” remain. This is because most of the areas affected by the schistosomiasis epidemic are economically challenged rural areas. Owing to the limited availability of local arable land and the fact that the labor force is mainly middle- and old-aged farmers who lack mechanical knowledge, there is some resistance to implementation of the strategy to “replace cattle with machinery” [29]. In addition, climate change, social factors, and other changes simultaneously affect the spread of schistosomiasis. For example, the construction of large-scale water conservancy projects, such as the construction of the Three Gorges project, changed the ecological environment of the reservoir and downstream areas, both of which have a direct impact on the habitat of snails [28,30,31]. Thus, the clusters of schistosomiasis began to reappear.

**Figure 6:**
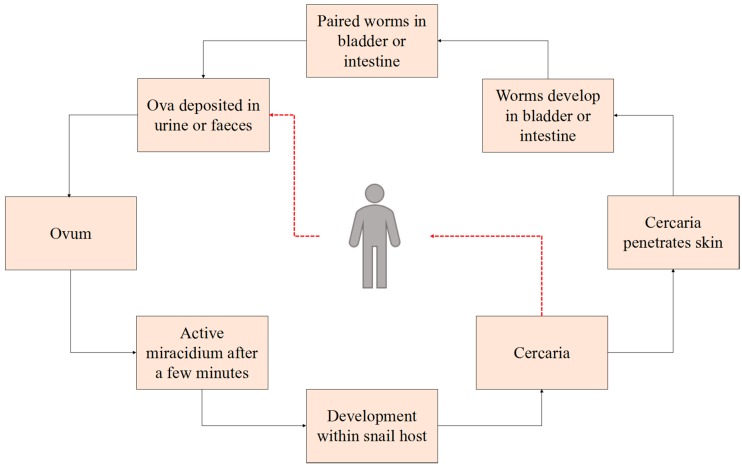
Disease transmission and life cycle of schistosomiasis.

The transmission of schistosomiasis is a complex process (Figure 6). It is closely associated with the propagation of *Schistosoma* and is largely related to temperature and the spread of intermediate snail hosts. The spread of snails is the main means of transmission and is related to vegetation coverage, land use patterns, soil quality, humidity, and other environmental conditions [20,32,33]. Factor analysis showed that land use, soil type, and temperature were all essential factors during the 6 years under investigation. Previous studies have shown that land use has a major impact on the distribution of intermediate host snails of schistosomiasis only [34,35,36,37]. In addition, significant differences have been reported in *Oncomelania* density and incidence among different land use types. The *Oncomelania* density and live snail occurrence tend to be lowest in forested areas, followed by dry land, and tend to be higher in paddy fields, floodplains, barren slopes, and ditches. Moreover, based on linear regression analysis of the ratio of land use in Jianghan Plain and the average density of village-level snails, Chang et al. [38] found that land use considerably affects snail distribution.

In addition, soil is necessary for the survival of snails and has a considerable influence on the spread of schistosomiasis. Previous studies have shown that soil texture (clay percentage, silt percentage, and sand percentage) can all affect the distribution area and density of snails in Hubei Province [33,39]. In the present study, we found that the soil type was also an important factor that influenced schistosomiasis; however, soil texture was a relatively weak factor, as compared to the other factors. Ambient temperature is another critical ecological factor for snails. It can affect their growth, development, reproduction, and distribution both directly and indirectly. Snails maintain a relatively narrow temperature range that is suitable for their physiological needs, between 20 and 30 °C. Environments that are either too cold or too hot are not conducive to their activity, reproduction, and even life expectancy [33].

It is worth noting that the influence of different factors on schistosomiasis varied with time. We analyzed the most important factors that affected the incidence of schistosomiasis, such as temperature, land use, and soil type. We found that the average LSTD, average LSTN, and the average TDN showed fluctuations in trends from 2007 to 2012. Studies [40] have shown that changes in temperature do not only affect the infection rate of *Oncomelania* snails, but within a certain range, temperature can also directly affect the growth rate of schistosome worms in *Oncomelania*. Furthermore, temperature changes could potentially affect mammals with schistosomiasis. When there is a change in temperature and the human infection rate has been affected, the distribution of schistosomes may also vary from year to year depending on the temperature. Thus, variations in the infection rate of each subarea might differ, resulting in different final q-values. Furthermore, the annual effect of temperature on schistosomiasis is not uniform. Although the land use data, soil type data, and soil texture data (silt percentage, sand percentage, and clay percentage) used in the present study were consistent from 2007 to 2012, differences in the magnitude of impact on schistosomiasis in different years were still observed. We believe that this is mainly due to changes in the infection rate, which declined from 2007 to 2012. Even if an impact factor shows no change, once the infection rate changes, the explanatory power of the various factors will also change.

In addition, we found that the distance between the administrative village and the water system had little influence on the infection rate in human schistosomiasis. Population contact with a contaminated water supply in an epidemic area is an important link in the spread of schistosomiasis [40]. Residents’ contact with contaminated water is affected by geographical, social, economic, cultural, and general living habits and tends to be seasonal. Quantitative observation of exposure of the residents to the water supply in endemic areas revealed that the chances of exposure to contaminated water during the busy months of May to July were the greatest. During the summer, various activities such as water bathing, catching fish, etc. are the main means of contact with contaminated water. However, owing to the development of comprehensive prevention and control measures for schistosomiasis, such as health education, altering planting patterns, machine-assisted cattle, and a safer water supply [41], the chances of villagers’ contact with contaminated water has been reduced.

It should be pointed out that this paper identified only the temporal and spatial clustering characteristics of a number of schistosomiasis cases. In addition, it analyzed some environmental factors of infection rates in human schistosomiasis. However, some limitations must be acknowledged. The spatiotemporal spread of schistosomiasis is a complicated, dynamic process (Figure 6) based on the relationship of infectious sources to media and subsequent host propagation [34]. This process is affected by the natural environment, social and human activities, and other factors. Because of the diversity of geographic elements, the Geodetector model is incapable of detecting all indicator factors and might reflect an appropriate type or range of environmental indicator factors that is inconsistent with the actual situation. Moreover, in comparison to quantitative factors, the use of qualitative factors can be subjective, and the values thereby derived are determined by their nature or attributes. Furthermore, arbitrary methods of discretization might not accurately characterize actual associations between risk factors and a health outcome; thus, some prior knowledge would be helpful in discretizing quantitative variables [42].

## 5. Conclusions

The present study systematically demonstrated the spatiotemporal clustering patterns of schistosomiasis-infected cases. Environmental factors associated with the rates of schistosomiasis infection in humans were detected and analyzed through use of the Geodetector and application of SaTScan statistics. In order to fundamentally eliminate the harm posed by schistosomiasis, prevention and control efforts should be focused on the following areas: the southwestern region of Qianjiang City, the vast majority of areas of the Shashi District, Jiangling County, Jianli County, and Gong’an County. Furthermore, temperature, land use, and soil type are the main factors responsible for schistosomiasis prevalence in Jianghan Plain, and most interactions among the risk factors enhance their singular effects.

## Figures and Tables

**Table 1:** Spatial analysis scanning for clusters with large populations of schistosomiasis infections using Kulldorff’s spatial scan statistic.

Year	Number of Clusters	Most Likely Cluster						
		Number of Villages	Latitude	Longitude	Radius (km)	LLR	RR	*p*-Value
2007	109	259	30.201	112.575	22.760	10,609.116	2.448	0
2008	74	1135	29.834	112.450	62.666	12,127.637	2.118	0
2009	88	996	29.840	112.424	59.289	8431.589	1.979	0
2010	70	1106	29.846	112.494	60.754	10,308.459	2.261	0
2011	68	1147	29.697	112.539	72.155	8138.424	2.112	0
2012	42	1147	29.697	112.539	72.155	7303.115	2.174	0

LLR, log likelihood ratio; RR, relative risk.

**Table 2:** Space–time analysis of clusters with large populations of schistosomiasis infections using Kulldorff’s spatial scan statistic.

Cluster	Number of Clusters	Number of Villages	Latitude	Longitude	Radius (km)	Time	LLR	*p*-Value
Most likely cluster	1	1014	30.755	112.970	87.938	2007	1404.203	0
Secondary likely clusters	2	116	30.290	111.741	30.583	2007	439.265	0
	3	500	29.689	112.274	47.596	2010–2012	395.753	0
	4	23	30.052	113.885	8.545	2010–2011	153.546	0
	5	4	30.110	113.723	5.146	2009	82.678	0

LLR, log likelihood ratio.

**Table 3:** Results of factor detector analysis.

Year	Land Use (%)	Soil Type (%)	Average NDVI (%)	Silt (%)	Sand (%)	Clay (%)	DER (%)	Average LSTD (%)	Average LSTN (%)	Average TDN (%)
2007	3.37	1.93 *	0.71	0.95	1.00	1.04	0.08 *	0.34 *	5.48	5.71
2008	4.69	2.46	0.25 *	0.15 *	1.38	1.08	0.51	0.63	1.61	1.59
2009	5.5	4.26	0.76	0.26 *	2.15	2.08	0.58	2.15	2.86	0.46 *
2010	5.13	4.06	0.81	0.25 *	2.44	1.84	0.58	4.56	10.53	1.81
2011	4.05	3.09	0.34 *	0.16 *	2.64	1.81	0.34	2.98	2.66	3.83
2012	4.28	2.67	0.43	0.57 *	2.19	2.10	0.06 *	0.76	6.50	4.01

Average NDVI, average normalized different vegetation index; DER, distance from the endemic village to the river; average LSTD, average land surface temperature at daytime; average LSTN, average land surface temperature at night; average TDN, average day and night temperature; * denotes *p*-value > 0.05.
